# Brazos Valley Medical Association

**Published:** 1897-06

**Authors:** W. B. Briggs

**Affiliations:** Secretary


### Brazos Valley Medical Association—Meeting at Cameron, Texas, May 11th and 12th, 1897.

Meeting called to order in Odd Fellows' Hall at 2 p. m. by President H. W. Cummings, M. D.

Address of welcome was delivered by Mr. R. M. Campbell on behalf of the mayor of Cameron, in which the keys of the city were turned over to the Association. This was responded to in a most pleasant manner by President Cummings.

The following physicians were elected to membership:

Drs. D. L. Peeples, of Navasota; J. C. Holman, of Franklin; R. R. Fountaine, of Jones' Prairie; L. E. Moore, of Buckholts; R. K. Ferguson, of Yarrelltown; D. L. Treadaway, of Lilac;

G. C. McCloud, of Buckholts; M. K. Lott, D. Monroe, E. N. Shaw, of Cameron, R. W. Nobles, of Temple; A. J. Ellzey, of Lilac; C. M. McClorty, of Dime Box; J. M. Dollar, of Gauze; A. C. Scott, of Temple.

The following papers were read:

"Malarial Hæmaturia," by Dr. Rriggs, of Easterly.

"Third Stage of Labor," by Dr. B. F. Watkins of Bryan.

An invitation was extended by Dr. T. A. Pope to the Asiociation to dine at his residence at 6:30 p. m., which was accepted.

Meeting adjourned to 8:30 a. m., May 12th.

The dinner at Dr. Pope's could not be surpassed. The tables were loaded with choicest delicacies, and at each of the many tables were seated ladies and gentlemen who, judging from smiling faces and happy humor, enjoyed the feast to the fullest extent. The occasion will never be forgotten.

Meeting called to order at 8:30 a. m., when the following papers were read:

"Inflammation," by Dr. D. L. Peeple, of Navasota.

"Has Medicine of the Past Advanced?" by Dr. M. K. Lott, of Cameron.

"Infant Feeding—Its Results," by Dr. G. M. Abney, of Franklin.

"Diseases Incident to Pregnancy," by Dr. J. W. Hudson, of Milano.

"La Grippe," by Dr. W. W. McDonald, of Easterly, read by Dr. W. B. Briggs.

"Dysentery," by Dr. A. J. Ellzey, of Lilac.

"Eczema," by Dr. W. Nobles, of Temple.

Perinæorrhaphia," by Dr. A. C. Scott, of Temple.

"Membranous Laryngitis," by Dr. J. P. Oliver, of Caldwell.

All papers were thoroughly discussed and ordered printed in all the medical journals of the State.

The election of officers resulted as follows:

President—Dr. G. M. Abney, of Franklin.

First Vice-President—Dr. D. L. Peeples, of Navasota.

Second Vice-President, Dr. R. K. Fontaine, of Jones' Prairie.

Secretary—W. B. Briggs, of Eastley.

Treasurer—Dr. W. W. Greer, of Cameron.

The following form the Judicial Council: Drs. M. K. Lott, of Cameron; M. L. Langford, of Bailyville; J. P. Sessions, of Rockdale; Geo. R. Tabor, of Bryan; A. J. Ellzey, of Lilac.

The query committee consists of Drs. H. W. Cummings, of Hearne; J. W. Hudson, of Milano; E. N. Shaw, of Cameron.

The place and time selected for the next semi-annual meeting was Navasota, second Tuesday in November, 1897. Dr. D. L. Peeples suggested Navasota, and was complimented.

A beautiful poem was composed and presented to the Association by Mrs. Augusta Antony, of Cameron. This poem was impressively read by Dr. Cummings, and a vote of thanks was tendered Mrs. Antony for the beautiful sentiments contained therein, and the poem was ordered printed in the *Cameron Herald.*

A vote of thanks was tendered to the physicians, Mr. Geo. A. Thomas and the other druggists, and to citizens for the feast tendered the members of the Association, and to the ladies for their presence at the residence of Dr. T. A. Pope.

A vote of thanks was also tendered the Odd Fellows, and to the members of the Methodist church for the use of their buildings.

Many ladies were present at the reading of the papers by Dr. Abney and Scott, and they were kindly thanked for their presence, and invited to return at pleasure.

On motion the meeting adjourned.

W. B. BRIGGS, M. D., Secretary.

---

### To the Brazos Valley Medical Association.

On the heights of Mount Olympus,
In the classic days of yore,
All sought the heathen deities
And worship on their shore.

'Twas then the gods made largess
In mystic courts, we're told,
And science slept embryo
E'er the earth had waxened old.

And Apollo's son, most favored,
Was given the goodly part,
And man has ever blessed him,
King of the healing art.

Now, to his wise disciples,
A nervy, brainy guild,

We pour our best libations
  Till every beaker's filled.

With sweetest draughts of nectar,
  And words most kind and true,
Go bring from rocks ambrosia
  Kissed by the sacred dew.

Lay it on this proud day's altar,
  While tuneful greetings ring,
On the fragrant air our welcome
  To the honored guest we bring.

From the East in the Brazos Valley,
  From the North, the South, the West.
And gather in love fraternal
  Our country's noblest, best,

And the old historic city
  Swings all her gates ajar
As Cameron bids thrice welcome
  These friends from near and far.

Ye potent seers of learning,
  Subtle and all supreme,
Upon thy tender sympathies
  Our frail, weak bodies lean.

Thy hands hold secrets hidden
  From every human ken;
Thine eyes behold the skeletons.
  In the hearts of women, men.

Often thy skill and knowledge
  Old Charon's boat defies,
And from the couch of suffering
  The dark-winged angles flies.

We call thee not omnipotent,
  That would dishonor God,
Whose sweet, infinite mercy gave
  To doctors Aaron's rod.

That they might smite the rock afresh,
  While from the fountains flow

The oil that brings surcease to pain
Of mortals here below.

Then here's to Æsculapius,
The founder of the art,
We raise our voice to bless
Thy student's faithful heart.

May his brave spirit be in touch
With precious gifts divine,
And we'll all bless the doctor's name
Through ringing change of time.

*Augusta Antony, in Cameron Herald.*

